# Three approaches to glucose monitoring in non-insulin treated diabetes: a pragmatic randomized clinical trial protocol

**DOI:** 10.1186/s12913-017-2202-7

**Published:** 2017-05-25

**Authors:** Laura A. Young, John B. Buse, Mark A. Weaver, Maihan B. Vu, April Reese, C. Madeline Mitchell, Tamara Blakeney, Kimberlea Grimm, Jennifer Rees, Katrina E. Donahue

**Affiliations:** 1 Division of Endocrinology, Department of General Internal Medicine, School of Medicine, University of North Carolina at Chapel Hill, 8025 Burnett Womack Building, Campus Box # 7172 UNC-CH, Chapel Hill, NC 27599-7170 USA; 2 Cecil G. Sheps Center for Health Services Research, University of North Carolina at Chapel Hill, Chapel Hill, NC USA; 3 UNC Gillings School of Global Public Health, Chapel Hill, USA; 4 Department of Family Medicine, School of Medicine, University of North Carolina at Chapel Hill, Chapel Hill, NC USA; 5 Center for Health Promotion and Disease Prevention, UNC Chapel Hill, Chapel Hill, USA; 6 North Carolina Division of Public Health, Chapel Hill, USA

**Keywords:** Type 2 diabetes, SMBG, Self-monitoring of blood glucose, Glycemic control, Pragmatic clinical trial

## Abstract

**Background:**

For the nearly 75% of patients living with type 2 diabetes (T2DM) that do not use insulin, decisions regarding self-monitoring of blood glucose (SMBG) can be especially problematic. While in theory SMBG holds great promise for sparking favorable behavior change, it is a resource intensive activity without firmly established patient benefits. This study describes our study protocol to assess the impact of three different SMBG testing approaches on patient-centered outcomes in patients with non-insulin treated T2DM within a community-based, clinic setting.

**Methods/Design:**

Using stakeholder engagement approach, we developed and implemented a pragmatic trial of patient with non-insulin treated T2DM patients from five primary care practices randomized to one of three SMBG regimens: 1) no testing; 2) once daily testing with standard feedback consisting of glucose values being immediately reported to the patient through the glucose meter; and 3) once daily testing with enhanced patient feedback consisting of glucose values being immediately reported to the patient PLUS automated, tailored feedback messaging delivered to the patient through the glucose meter following each testing. Main outcomes assessed at 52 weeks include quality of life and glycemic control.

**Discussion:**

This pragmatic trial seeks to better understand the value of SMBG in non-insulin treated patients with T2DM. This paper outlines the protocol used to implement this study in fifteen community-based primary care practices and highlights the impact of stakeholder involvement from the earliest stages of project conception and implementation. Plans for stakeholder involvement for result dissemination are also discussed.

**Trial registration:**

ClinicalTrials.gov NCT02033499, January 9, 2014.

## Background

Over the past decade the value of routine daily self-monitoring of blood glucose (SMBG) in patients with type 2 diabetes (T2DM) not treated with insulin has been contentiously debated [1–8]. Proponents postulate that testing promotes better awareness of glucose levels, leading to improvements in diet and lifestyle. When test results are shared with health care providers, it is argued, there is also the potential for more timely treatment modification. Competing arguments point to the costs of SMBG, both in terms of supplies (test strips and meters) and time, as well as discomfort and potentially quality of life. As a result no clear consensus exists regarding SMBG monitoring in non-insulin treated patients with T2DM.

In an attempt to make SMBG more convenient and patient-centric, much effort has been placed in improving SMBG technology [9]. Early studies that marry technology and SMBG been mixed, with some showing a benefit on glycemic control and others showing no benefit [1, 10]. Few trials have pragmatically assessed the utility of SMBG monitoring in real-life settings, like the busy primary care practice where the majority of non-insulin treated patients with T2DM are managed. With patients taking a more directive role in their health care, a large focus is being placed on how usable these glucose-monitoring tools are in the real world. Few studies have focused on the health care provider side of this issue. In fact, studies of ‘enhanced’ SMBG, where both the patient and the provider were engaged in SMBG interpretation, found A1c reductions along the magnitude of 0.5% [4, 11–13]. As additional ‘enhanced’ intervention SMBG studies have been added to the literature [13, 14], more recent reviews and meta-analyses have drawn conclusions more in favor or testing [4, 15]. This pattern suggests that, for SMBG to be an effective self-management tool in non-insulin treated T2DM, the patient and the health care provider must both actively engage in performing, interpreting, and acting upon the SMBG values.

Given these unanswered questions regarding the impact of SMBG on patient quality of life and other patient reported outcomes, there is a growing interest by patients and other stakeholders who are looking for data that will help them make better, informed decisions about their self-care when no standard of care exists and providers make recommendations based upon their experience and preferences, like SMBG monitoring in T2DM. Our overarching goal is to answer the following questions: Is SMBG testing effective for people with non-insulin treated T2DM in terms of either A1c or quality of life (QOL)? We will also examine outcomes from SMBG in patients with different baseline characteristics. Our primary outcomes include change in glycemic control over 52 weeks and change in QOL over 52 weeks. The purpose of this paper is to describe our study methods and the unique aspects of stakeholder engagement that have been employed during the design and implementation of the study.

## Methods/Design

### Study overview

The overarching goal of this proposal is to assess the impact of three different SMBG testing approaches on patient-centered outcomes in patients with non-insulin treated T2DM within the real-world, clinic setting. In this pragmatic trial, 450 patients will be randomized to one of the following three SMBG testing regimens: 1) no SMBG testing, 2) once daily SMBG testing with standard patient feedback consisting of glucose values being immediately reported to the patient through the glucose meter, and 3) once daily SMBG testing with enhanced patient feedback consisting of glucose values being immediately reported to the patient PLUS automated, tailored feedback messaging following each SMBG testing event delivered to the patient through the glucose meter.

### Recruitment

We will recruit 450 patients from primary care practices within the central North Carolina area. Patients will be randomized to one of the three study arms: 1) no SMBG testing; 2) once daily SMBG testing with standard patient feedback which includes the current glucose value; and 3) once daily SMBG testing with enhanced patient feedback which includes the current glucose value and a personalized message about the current glucose value. We have elected to use an FDA approved, cellularly-enabled glucometer device given its capabilities to deliver messaging to patients in real time. Participants in both the standard feedback and enhanced feedback groups will all receive the glucometer. Patients will be followed for 1 year. The first two study groups represent SMBG testing approaches commonly utilized in clinical practice, while the third incorporates cutting edge glucose monitoring tools now on the market. During routine clinic visits, health care providers will be guided to modify therapies based upon American Diabetes Association (ADA) guidelines, which focus on A1c values and SMBG values if available. Patients in groups 2 and 3 will also receive training in obtaining and interpreting SMBG values. SMBG values will be systematically evaluated at routine clinic visits.

### Stakeholder Engagement

As part of this grant, stakeholders were encouraged to play a role in providing perspective to the research questions and methodologies chosen, and patient related outcomes. There were several stakeholders that provided a critical role in providing care advocacy and education for persons with diabetes in North Carolina, which included patient groups community members at risk individuals policy makers providers industry and professional organizations. Given all of these stakeholder groups involved in the area of diabetes we chose a broad yet appropriately sized swath of this community. This included the North Carolina Diabetes Advisory Council, a patient advisory board, a community advisory board, health care providers, American diabetes Association representative, National Diabetes Education Program representation, as well as patients involved in the Diabetes registry and Glucometer manufacturers. Two primary methods were planned for continuing collaboration with these stakeholder groups. This included attending regularly scheduled meetings the larger organizations on a pre-stipulated basis and holding key stakeholder teleconferences with a small group (~10) of representative stakeholders.

### Patient identification and recruitment

A multi-pronged approach will be utilized to identify study participants. First, patients who have non-insulin treated T2DM will be identified using the Carolina Data Warehouse (CDW) or by chart review in the participating practice. Patients will receive an invitation letter by mail, signed by their health care provider describing the study. Any patient not interested in participating will be able to opt out of further contact by returning a post card or calling a toll-free number. If no opt out call is made within 1 week of sending the letter, telephone contact will be attempted on up to three different occasions at three different times of day. Second, we will place recruitment flyers within the participating practices that will be visible in waiting areas and exam rooms. Clinic staff will be encouraged to discuss the study with potentially eligible patients during routine clinic activities (i.e. while collecting vital signs and during check-in or check-out). The UNC field coordinators will communicate regularly with practice staff regularly regarding potentially eligible

All potentially eligible patients will be screened for eligibility by phone call. Overall inclusion and exclusion criteria are listed in Table 1. The call will take about 15 min, during which a member of the study team describes the study, answer questions, and conducts a short set of simple screening questions. Eligible interested patients complete an assessment visit with a research coordinator. To decrease patient burden and further engage participating practices, assessment visits occur at the patient’s primary care office. Assessments are separate from their appointment with their primary care provider, and may or may not occur on the same day as a regularly scheduled clinic visit, though for patient convenience we make every effort to coordinate the assessments with a regular clinic visits. During these assessments, the research coordinator reviews the study details in greater depth, verify all inclusion and exclusion criteria, and obtain written informed consent.

**Table 1:** Monitor trial inclusion and exclusion criteria

Inclusion Criteria	Exclusion Criteria
T2DM diagnosis Age ≥ 30	Sees an endocrinologist or other diabetes specialist
An established patient at the participating practice	Use of insulin.
≥ 6.5% but ≤ 9.5%	Pregnancy
Willing to comply with random assignment into a study group	Plans to relocate in the next 12 months.
No history of significant issues with hypoglycemia	Has other conditions (e.g. renal or cardiovascular disease), factors (e.g. frailty) or comorbidities (e.g. cancer) that might put the patient at risk

### Data collection

#### Baseline

Eligible patients interested in enrolling for the study are subsequently scheduled for the initial visit with the study Field Coordinator. During this initial visit, the Field Coordinator reviews and obtains written informed consent from the patient. A signed copy is provided to the participant. After written informed consent has been received, blood samples are obtained along with height and weight. Standardized surveys are completed, and patients have the option of completing them independently or with the assistance of the Field Coordinator.

#### Randomization

**Fig. 1:**
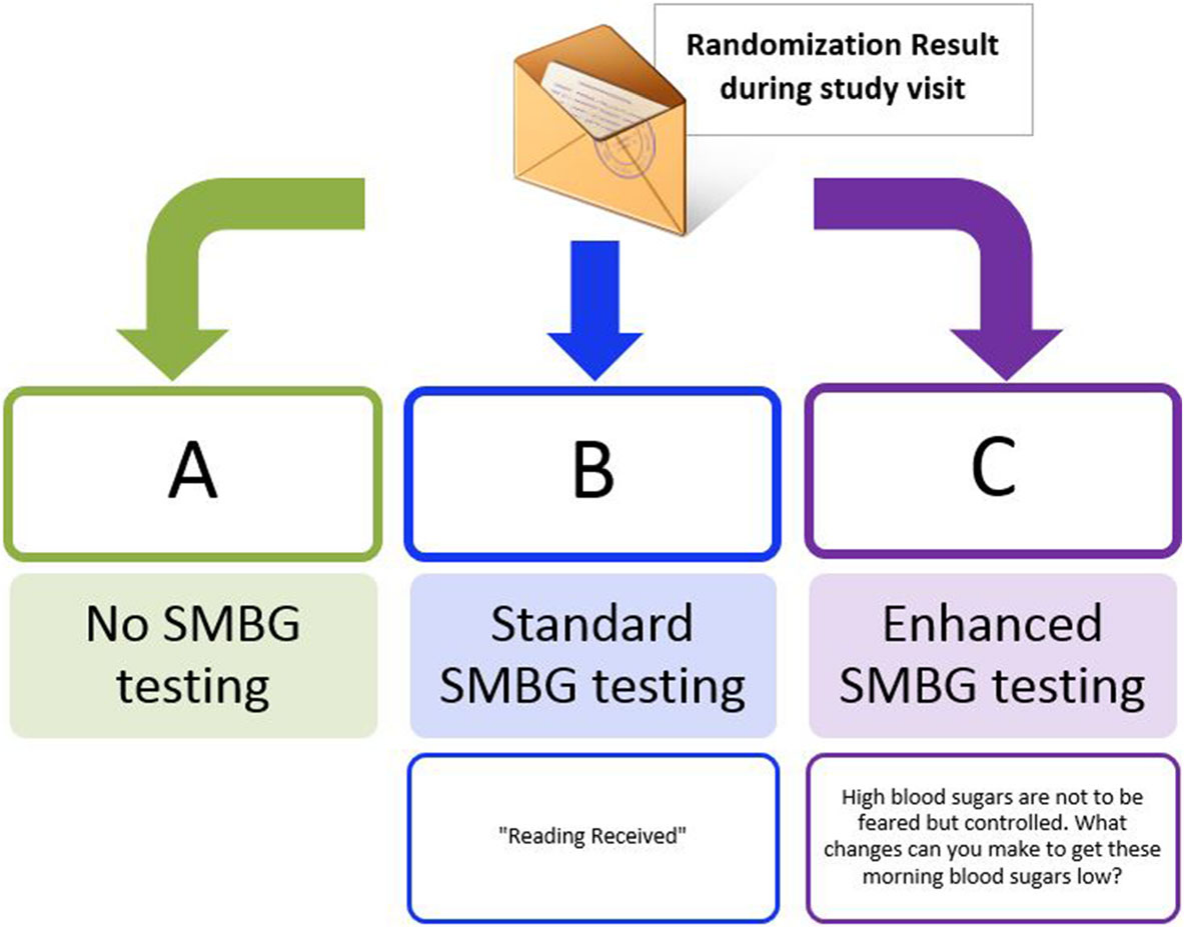
Study Arms

After providing informed consent, baseline A1c, and completing the baseline study interview, participants will be randomized to one of the three treatment arms using sequentially numbered, opaque, sealed envelopes. The allocation sequence is generated using computer-generated randomly permuted blocks of random sizes. The randomization is stratified by study practice. The Field Coordinator reviews the treatment assignment with the patient, using a standardized script, provide the initial training and supplies necessary for participation in the study and answer any remaining questions (Fig. 1).

#### 52 week follow-up

Patient will have blood drawn for a A1c. Patient participants will complete a follow-up interview that includes demographic, health history, and quality of life questions. Weight measurement will be collected.

#### Measures

The two primary outcomes of the study are change in A1c and change in health related quality of life (HRQOL). The A1c was chosen due to its use in prior studies and its standing as a measure on which: 1) providers evaluate patients; 2) payers evaluate providers; and 3) patients evaluate themselves and their providers [2]. Though HRQOL is also critically important to patients, few studies have rigorously examined the impact of SMBG on HRQOL. Based on these facts and the recommendations of our stakeholder groups, HRQOL is a co-primary outcome for this pragmatic trial. We will utilize the Short Form 36 (SF-36) to assess overall quality of life. We will use the physical component score (PCS) and the mental component score (MCS), with scores standardized to a normal distribution (mean = 50 and standard deviation[SD] = 10) [16]. It has been widely used and validated in medical studies generally and diabetes studies in particular [17–20]. All primary and secondary outcomes are outlined in Table 2. Because the 52-week time point may not align with a scheduled primary care visit, we have defined this time point as 52 ± 6 weeks from the baseline visit.

**Table 2:** Primary and secondary patient-centered outcomes

	Clinical Outcomes	Time	Patient Reported Outcomes	Time
Primary	Glycemic control (A1c)	Baseline/52 weeks	Quality of life (QOL)	Baseline/52 weeks
Secondary	Hypoglycemia frequency	Baseline/52 weeks	DM-related QOL	Baseline/52 weeks
	Health care utilization	Baseline/52 weeks	DM self-care	Baseline/52 weeks
	Treatment regimen modification	Baseline/52 weeks	DM treatment satisfaction	Baseline/52 weeks
			DM-related self-efficacy	Baseline/52 weeks
			Patient-provider communication	Baseline/52 weeks

The following secondary endpoints will be collected from patient participants at baseline and 52-weeks. 1) Problem Areas in Diabetes (PAID) to assess psychological and social stress associated with diabetes [21–23], 2) Diabetes Symptom Checklist (DSC) to measure of diabetes-related symptom frequency and perceived severity during the prior month covering six symptom categories: hyperglycemic, hypoglycemic, cardiac, neuropathic, psychological, and vision-related [24–26] 3) Summary of Diabetes Self Care Activities (SDCA) survey to measure self-management activities (diet, exercise, blood glucose testing, foot-care, and smoking status and a supplemental medication adherence question [27], 4) the Diabetes Treatment Satisfaction Questionnaire (DTSQs) standard version to assess this diabetes treatment satisfaction [28], 5) Diabetes-Specific Self-Efficacy using the Diabetes Empowerment Scale Short Form [29], 6) Patient-Provider Communication using the Communication Assessment Tool [30].

#### Hypoglycemia Frequency

At the 52-week follow-up visit, participants are asked to report the number of hypoglycemic episodes that required intervention by a caregiver or by medical or paramedical personnel since enrolling in the study. For each episode, the participant will be asked whether EMS was called or led to an urgent care clinic or emergency department visit, or hospitalization. Each report is reviewed by the study principal investigators and co-investigator to determine whether the event is study related. A summary report is submitted to the UNC Institutional Review Board.

#### Health Care Utilization

Utilization outcomes will include inpatient, outpatient and emergency department utilization during the 52-week study period. UNC visits will be collected through the EHR while non UNC visits will be captured at the 52 week study visit via patient interview with the study coordinator.

#### Adverse Events

Any reported adverse events will be categorized as related or not related to the study intervention by the investigators. These events will be reported in real time to the principal investigators for confirmation and review. Regardless of causality, all unanticipated Serious Adverse Events will be reported to the IRB for review.

#### Qualitative Assessment

To gain a deeper understanding of patients’ and health care providers’ experiences with each of the SMBG testing approaches, including facilitators and barriers to dissemination, we will conduct focus group discussions at the UNCPN practices.

##### Qualitative Assessment of Patient Outcomes

Individual phone interviews will be conducted by researchers skilled in qualitative data collection. At each participating practice, we will interview approximately 6 patients who have completed the study. Two patient participants from each arm will be recruited for each of the 13 practices (*N* = 78 patients). Every effort will be made to recruit a range of high and low engagement for testing arms. Interviews will last approximately 30–45 min and will include these topics: impressions of and experience with SMBG prior to and during the trial, including using a wireless glucometer and messaging system, and impressions of using the downloaded SMBG reports at clinic visits, interactions with providers about diabetes care before and during the study, and strategies for dissemination and communication. All participants will review, sign, and be given a copy of the IRB-approved informed consent document specific to the focus groups.

##### Qualitative Assessment of Health Care Provider Outcomes

Focus groups (one per practice) with 5–10 physicians and nursing staff will also be conducted. by researchers skilled in qualitative data collection. Focus groups will be conducted once at least 80% of that practice’s enrolled study participants will have completed their 52-week follow-up visit. Key discussion topics will include: impressions regarding the usefulness of SMBG summary reports and accompanying treatment algorithm; experience with recommending SMBG for patients with NIT DM prior to and during the trial (and for both enrolled and non-enrolled patients); and perceived benefits/problems arising from being a practice site for this study. All participants will review and sign IRB-approved informed consent documents prior to participation. We will also collect age, gender, race/ethnicity, full-time/part-time employment status, years in practice, and area of practice.

## Sample Size

We desire high power for our primary between-arm comparison as well as reasonable power to detect an important effect modifier, should one exist. In a recent Cochrane Review, the estimated mean 12-month differences between SMBG and control groups were−0.13 and−0.52%, respectively, for patients diagnosed more than and less than 1 year prior to the study [1]. Assuming approximately equal enrollment of newly diagnosed and long term patients, this implies a mean difference of−0.325%. Based on results from one of the larger and better quality trials conducted to date, we assume the standard deviation for ΔA1c would be 0.8% [4]. We assume no more than 10% loss to follow-up. Given these assumptions, randomizing 150 patients per group would provide at least 90% power for the primary comparison for A1c at the 0.05 significance level. This same sample size would provide at least 80% power for the primary comparison if the true standard deviation were as high as 1.0%. For comparing mean change in HRQOL between groups, we will use the physical and mental component scores of the SF-36, both of which range from 0 to 100. Because we are interested in two different aspects of QOL, we will apply a Bonferroni correction and assess each comparison at the 0.025 level. Based on observed results from the ZODIAC-17 SMBG study, we conservatively assume that the standard deviation for the change scores for either component will be 10 points [2]. Under these assumptions, randomizing 150 patients per group with no more than 10% loss will provide at least 80% power to detect an overall difference between groups if the mean difference between the highest and lowest groups is at least 4 points.

## Data Management and Statistical Analysis Plan

All data will be systematically coded and double-entered into a data system stored on a secure server according to institutional, state, and federal policies. Contact information was obtained from electronic health records and stored on a secure, password-protected server with access limited to study staff and will be destroyed after 5 years as required.

A detailed statistical analysis plan, finalized prior to enrolling the first patient, is available from the authors upon request; here, we provide key elements of that plan for analysing the co-primary outcomes. The biostatistician and principal investigators are blinded to treatment assignments, but the study field staff are not blinded. For primary analyses, all randomized patients will be analysed according to their randomized group regardless of the extent to which they performed SMBG (intention-to-treat). Missing 52-week outcome data will be ignored for the primary analyses. First, we will compare change in A1c from baseline through 52 weeks across the three randomization groups using an analysis of covariance (ANCOVA) conducted at the 0.05 significance level. This ANCOVA model will control for baseline A1c, prior use of SMBG, duration of T2DM, baseline anti-hyperglycemic treatment, age, race/ethnicity, health literacy, and number of baseline comorbidities. If the overall null hypothesis of no difference between the three groups is rejected, we will compare each SMBG group to the no testing group separately using the Dunnett-Tamhane Step-Up procedure for multiple comparisons to control the family-wise error rate at 0.05 (69). We will also conduct a contrast test comparing the average of the two SMBG groups to the no testing group at the 0.05 level. Similar ANCOVA models will compare change in SF-36 physical and mental component scores between groups, controlling for the same baseline variables as for A1c. Because we are examining two aspects of HRQOL, we will apply a Bonferroni correction and assess each comparison at the 0.025 level. We will not adjust for multiple comparisons due to the co-primary endpoints of A1c and HRQOL. In addition, we will assess for potential effect modification for each of the baseline variables included in the models by adding appropriate interaction terms to the ANCOVA model one at a time. Each interaction term will be tested separately at the 0.05 significance level. Only if the associated interaction term is significant, similar contrasts to those described above will be assessed within the relevant subgroups. Similar ANCOVA methods will be used to compare groups on each of the secondary outcomes. These tests will each be conducted at the 0.05 significance level with no adjustments for multiple comparisons.

### Baseline Statistics

Table 3 highlights the baseline statistics of the 450 subjects that have been recruited into the study.

**Table 3:** Baseline interview

	Mean (sd)	Min	Max	N (%)	# Missing
Demographics					
Age (years)	60.5 (11.5)	30.8	91.8		
Male				207 (46.0)	
Race					
American Indian or Alaskan Native				3 (0.7)	
Asian				9 (2.0)	
Black or African-American				148 (33.0)	
Native Hawaiian/Pacific Islander				1 (0.2)	
White				279 (62.3)	
Other (African, Filipino, Hispanic)				3 (0.7)	
Multiracial (1 = white + other, Indian)				5 (1.1)	
Refused (e.g., human race)					2
Ethnicity					
Latino/Hispanic				8 (1.8)	1
Education					
Less than high school graduate				25 (5.6)	
High school graduate or GED				107 (23.8)	
Some college or associate’s degree				164 (36.5)	
4-year college degree				92 (20.5)	
Graduate degree				61 (13.6)	
Refused					1
Marital status					
Married				292 (64.9)	
Widowed				37 (8.2)	
Living together				4 (0.9)	
Single				64 (14.2)	
Separated				7 (1.6)	
Divorced				46 (10.2)	
Comorbidities					
# comorbidities	3.4 (1.8)	0	10		
Heart disease				86 (19.2)	1
High blood pressure				340 (75.6)	
Lung disease				30 (6.7)	
Stroke				29 (6.4)	
High Cholesterol				299 (67.0)	4
Kidney disease				32 (7.1)	
Liver disease				18 (4.0)	
Anemia or other blood disease				61 (13.6)	
Cancer				76 (16.9)	
Depression/anxiety				139 (30.9)	
Arthritis				210 (46.8)	1
Chronic back pain				112 (24.9)	
Autoimmune disease				14 (3.1)	
Stomach or bowel disease				83 (18.4)	
Healthcare utilization					
Have you seen your primary care provider?	3.7 (2.4)	1	30		
Have you gone to an urgent care clinic?	0.5 (1.1)	0	12		
Have you been seen in the Emergency Room	0.4 (1.1)	0	12		
Have you been hospitalized overnight?	0.2 (0.5)	0	6		
Have you had someone call EMS for you?	0.1 (0.4)	0	5		
DM-related					
Nephropathy or other diabetes-related kidney disease				11 (2.4)	
Diabetic retinopathy or other diabetes-related eye disease				20 (4.4)	
Neuropathy or other diabetes-related nerve damage				65 (14.4)	
Years with T2DM diagnosis	8.2 (7.6)	0.1	50.0		
# Testing = Yes				338 (75.1)	
If yes, how often: at least daily				129 (38.2)	
If yes, how often: not daily, but > 1X/week				117 (34.6)	
If yes, how often: a few times a month				50 (14.8)	
If yes, how often: once a month				21 (6.2)	
If yes, how often: less than once a month				21 (6.2)	
If yes, how often told: at least daily				187 (55.5)	
If yes, how often told: not daily, but > 1X/week				53 (15.7)	
If yes, how often told: a few times a month				14 (4.2)	
If yes, how often told: once a month				2 (0.6)	
If yes, how often told: < once a month				1 (1.2)	
If yes, how often didn’t know				77 (22.9)	
If Testing = No, ever tested?				78 (69.6)	
If yes, at least daily				55 (70.5)	
If yes, not daily, but > 1X/week				10 (12.8)	
If yes, a few times a month				3 (3.9)	
If yes, once a month				1 (1.3)	
If yes, less than once a month				9 (11.5)	
If yes, how long did you test: <= 1 month				8 (10.3)	
If yes, how long did you test: <1 year >1X/month				35 (44.9)	
If yes, how long did you test: 1 year				12 (15.4)	
If yes, how long did you test: > 1 year < 5 years				17 (21.8)	
If yes, how long did you test: > = 5 years				6 (7.7)	
# Testing = No, why not?				112 (24.9)	
Not told to test				50 (44.6)	
Costs				18 (16.1)	
Pain				8 (7.1)	
Disruptive				4 (3.6)	
Does not understand why should test				7 (6.25)	
Constant reminder of DM				10 (8.9)	
Other reason				30 (26.8)	
Medications and BMI					
# of prescription medications	6.2 (3.4)	0	17		
# of DM medications	1.4 (0.9)	0	5		
BMI (max weighs 523 lb)	34.3 (7.7)	20.8	75.0		
Primary Endpoints					
Hemoglobin A1c (%)	7.6 (1.1)	5.7	13.1		
SF36—Physical Health	47.4 (8.9)	10.4	63.0		
SF36—Mental Health	53.3 (9.2)	9.5	68.0		
Secondary Endpoints^a^					
Diabetes Empowerment Scale—Short Form	4.3 (0.5)	1.1	5		1
Diabetes Symptom Checklist—Revised	20.4 (21.4)	0	107.2		
Diabetes Treatment Satisfaction—satisfaction	31.8 (5.1)	6	36		6
Diabetes Treatment Satisfaction—perceived control	2.7 (2.6)	6	12		23
Problem Areas in Diabetes	13.1 (16.2)	0	82.5		
Summary of Diabetes Self-Care Activities (SDSCA) (# of days in last 7)					
General Diet	4.3 (2.2)	0	7		1
Special Diet	4.1 (1.8)	0	7		
Carb spacing	4.1 (2.4)	0	7		
Exercise	2.9 (2.2)	0	7		
Blood glucose testing (only # times tested)	2.6 (2.8)	0	7		1
Foot care	3.2 (2.5)	0	7		
Communication Assessment Tool—MD items	4.5 (0.7)	1.5	5		1
Communication Assessment Tool—staff item	4.6 (0.7)	1	5		
Health Literacy					
Newest Vital Sign	3.8 (2.0)	0	6		2
Newest Vital Sign (% >4 = literate) (median = 4)				235 (61.8)	2

^a^Min/max may contain a decimal if missing data within the allowable limit were present

## Discussion

### Engaging Stakeholders

We designed the Monitor Trial using a pragmatic design that was informed by our stakeholders. Increasingly organizations like the Patient Centered Outcome Research Institute (PCORI) are encouraging the involvement of stakeholders in all aspects of research from study inception, design, implementation, analysis, and dissemination of results. Although the benefits and/or pitfalls of including stakeholders broadly has yet to be fully understood, this approach when executed effectively holds great promise for helping patients and providers make better, more informed choices about their health and healthcare. Below we describe how we have engaged our stakeholders to date and our plans for continued engagement.

The study team engaged a multitude of stakeholders during the inception of the study to ensure that issues and outcomes that mattered most to patients and heath care providers were fully considered. We engaged patients primarily via two avenues. First we attended two Patient Advisory Board (PAB) meetings to interact in real-time with patients, care givers and community members affected by diabetes. We also electronically surveyed patients from the UNC Diabetes Care Center Patient Registry, which at the time of the survey include over 2000 patients with diabetes across North Carolina. From these interactions we learned that there is a perceived direct link between SMBG and quality of life. Additionally the feedback we received confirmed our beliefs that quality of life is important to patients. Registry respondents reported that they would use SMBG more frequently if it held the potential to positively impact quality of life. During discussions with the PABs the topic of patient-provider communication arose. Insights from patients and caregivers highlighted the fact that patient-provider communication is an important issue and that avenues to improve this should be developed. It was viewed that consistently reviewing SMBG results together is an opportunity to build partnerships between patients and their health care providers. Registry respondents underlined current inconsistencies related to how SMBG values are utilized during clinic visits. Most (59%) reported that they believed it was important for medical providers to review SMBG values, but only 38% reported their health care providers always reviewed SMBG logs and provided feedback. Based upon this feedback we solidified the study team’s initial premise that for SMBG to be effective, both the patient and provider must be engaged in the process and as a result designed the study to include treatment recommendations that would be generated for each health care provider at the time of the clinic visit. These treatment recommendations are intended to serve as a guide to inspire discussion between patients and health care providers. To objectively capture this, we included a measure of patient-provider communication that is completed at baseline and end of study. We will be collecting qualitative data related to this topic at the conclusion of the study as well to provide greater insight.

Input from health care providers was also utilized during the design of this project. Early on the study team engaged the UNCPN membership during a large network-wide meeting. We discussed with the providers the varied recommendations made to patients related to SMBG monitoring in the real-world setting. Providers reported that the believed the primary question of SMBG monitoring effectiveness in T2DM to be an unsettled, high priority topic in primary care. This message was underscored by findings from the registry. Of the 62 registry respondents with T2DM not using insulin, 90% reported that they included SMBG as part of their self-care plan. Self-reported SMBG frequencies were: daily, 37.5%; several times per week, 37.5%; several times per month, 20%, and less than once per month, 5%. The other main take home point distilled from our conversations with providers related to the need for the proposed study to be “efficient” and minimally disruptive to the day-to-day clinic flow. This feedback guided the intervention development. Additional feedback during the planning phase of the MONITOR Trial was obtained by several other organizations outlined in Table 4.

**Table 4:** Stakeholders and the input in the study design

Stakeholders	Input provided	How it shaped our design
Diabetes Advisory Council/State Department of Health (DAC)	Consider health literacy issues of patients Policy subgroup would be useful to engage for this work	- Engaged the Center for Diabetes Translation and Research literacy core to join our team and assist with message tailoring - Tailoring algorithm that could be used in office - Brought in Diabetes Advisory Council as a policy subgroup
UNC Patient Advisory Board	Emphasize quality of life questions (e.g, Can I feel better or improve my ADLs?)	Added quality of life to outcomes
Greensboro Community Advisory Board	Important outcomes: Quality of life, hypoglycemia, health care service use, and patient empowerment. CMEs for providers, Query patient/provider community care	Hypoglycemia added as an outcome CME added for providers Added survey questions about patient-provider communication
Diabetes Center Patient Database	A1C is important in addition to Quality of life	A1C designated as a primary outcome
UNCPN Medical Directors	Testing is quite variable in real world clinical settings	Designed three-armed plan to address this reality and better respond to pragmatic patient issues.

We continue to engage the stakeholders during the grant process with periodic phone calls where they have provided input on that best methods of patient recruitment, dissemination opportunities, patient scripts used during the baseline interview process. The overall goal of involving various stakeholder groups is to have key people with a variety of perspectives involved in planning, oversight, analysis, and dissemination. We consider this collaboration essential for not only designing and conducting research study that is responsive to the needs and practical question non-insulin treated type 2 diabetes patient’s and caregivers daily but also to disseminating our findings.

### Clinical Implications

Self-monitoring of blood glucose in patients with diabetes has traditionally been considered to be a pillar of diabetes self-care. Scientific examinations of SMBG in T2DM have provided mixed results. An early epidemiological evaluation of the issue using a retrospective, longitudinal analysis showed that nonfatal micro- and macrovascular event rates along with fatal event rates were lower in individuals performing SMBG routinely as compared to those who were not [3]. A multitude of clinical trials followed. Several showed a significant benefit from SMBG testing on improving glycemic control [4–7], while others found no evidence of benefit [16]. In fact, some studies even suggested harm from routine SMBG in patients with T2DM, specifically, higher rates of depression and increased cost without accompanying benefits [31].

Given these mixed results, a series of meta-analyses and systematic reviews were conducted to investigate the benefit or lack thereof of SMBG on glycemic lowering in patients with T2DM [1, 9, 11–14]. While meta-analyses can be a useful way to assess the clinical effectiveness of an intervention, they are limited by the quality and comparability of the clinical trials included in the analyses. Issues of sample size, duration/details of the intervention, and patient characteristics (e.g., newly diagnosed vs. longer duration of disease; baseline A1c level), varied considerably across the available studies. Given these critical differences, it is perhaps not surprising that the results of the meta-analyses have also shown conflicting results. However, the overall conclusion has been that SMBG is likely not cost effective for this population of patients [1, 9, 12].

Perhaps most important to understanding these mixed results is the fact that the question being addressed by the studies is itself not consistent, falling generally into two camps: ‘simple’ SMBG and ‘enhanced’ SMBG. In studies testing simple SMBG, patients conducting SMBG were compared to patients who were not. In evaluations of ‘enhanced’ SMBG, intervention group patients and/or providers were given education or feedback such that they were better able to interpret SMBG results and use them in a meaningful way with regard to lifestyle changes and treatment modification. Among tests of ‘simple’ SMBG, A1c levels were reduced on average by 0.2%, an amount that was statistically significant in these studies, but of doubtful clinical significance [12, 13]. Studies of ‘enhanced’ SMBG found A1c reductions closer to 0.5% [7, 12, 15, 16]. As additional ‘enhanced’ intervention SMBG studies were added to the literature [13, 14], more recent reviews and meta-analyses have drawn conclusions more in favor or testing [4, 15]. This pattern suggests that, for SMBG to be an effective self-management tool in T2DM, the patient and the health care provider must both actively engage in performing, interpreting, and acting upon the SMBG values.

In an attempt to make SMBG more convenient and patient-centric, much effort has been place in improving the technology supporting SMBG. Like their non-technological predecessors the results from these early studies that marry technology and SMBG in an effort to make the process more convenient have been mixed, with some showing a benefit on glycemic control and others showing no benefit. With patients’ taking a more directive role in their health care, a large focus is being place on how usable these glucose-monitoring tools are in the real world. Generally speaking many of the applications need to focus on improved usability, perceived usefulness, and sustained adoption of the technologies [6]. Web-based applications, applications with easy-to-use mechanisms, and applications without location restrictions (can be used at home) have been most useful [7]. Furthermore, few studies have focused on the health care provider side of this issue. Allowing consistent, hassle free access to SMBG values for providers is an equally important part of the equation. In fact, studies of ‘enhanced’ SMBG, where both the patient and the provider were engaged in SMBG interpretation, found A1c reductions along the magnitude of 0.5% [7, 12, 15, 16]. As additional ‘enhanced’ intervention SMBG studies have been added to the literature [13, 14], more recent reviews and meta-analyses have drawn conclusions more in favor or testing [4, 15]. This pattern suggests that, for SMBG to be an effective self-management tool in non-insulin treated T2DM, the patient and the health care provider must both actively engage in performing, interpreting, and acting upon the SMBG values.

One issue often neglected in prior work is the fact that SMBG could impact patient quality of life (QOL) both positively and or negatively. Testing itself is a burden and could act as a constant reminder of one’s less than ideal health status [32]. On the other hand, testing may provide a sense of agency, improving a patients self of self-efficacy and hope for maximizing health and independence into the future [32]. A recent Cochrane Review identified only a handful of studies that had examined health-related quality of life (HRQOL), well-being, or patient satisfaction [1]. While these studies did not find clinically relevant differences in HRQOL for those who do or do not test, the review indicated future research was needed [1]. In one study, HRQOL initially decreased, but follow-up qualitative interviews showed that patients in the testing groups experienced an increased awareness of illness [4]. While both simple and ‘enhanced’ versions of SMBG were evaluated, the enhanced version included only training in the meaning of the results and encouragement to explore how lifestyle and dietary choices affect test values). Without more hands-on use of results (e.g., reports provided to the care provider), patients might have felt more ‘overwhelmed’ than empowered by the experience of testing. One of the key features of the MONITOR Trial is that we are focusing on HRQOL and other patient-centered aspects of SMBG and by the nature of the study design we are examining the impact of this self-care activity within the context of patient’s having actionable knowledge and improved opportunities for provider/patient collaboration.

While researchers and medical organizations debate the overall issue of the value of SMBG testing in NIT DM, patients face this choice daily and without adequate information as to its clinical or psychological outcomes. For some patients, their decision on testing will mirror that of their care provider. Yet more and more, patients play an active role in managing their own health. To some degree this is a necessary trend, because providers simply do not have sufficient time to provide intensive ongoing and comprehensive education and decision-making around diabetes self-management. Then too, even providers are looking for additional guidance on this question, including how to present options to patients and incorporate test results into care [7]. The fact that patients are taking a greater role in their health care is generally positive, because those who do so also improve their outcomes.

In order to make informed patient choices, patients and their providers need accurate, generalizable and meaningful information about the merits or demerits of SMBG testing. Because the existing research, though relatively extensive, has not yet met this important need, more research must be done on this issue. The Consensus Report of the Diabetes Technology Society provides a list of recommendations for future research relating to SMBG. In regard to the intervention itself, they recommend that it a) is linked to a structured program designed to facilitate behavior change, b) has A1c as a primary endpoint but include patient-centered endpoints, c) include encouragement and support, preferably in the form of personalized, automated feedback to patients in real time, d) takes advantage of telemedicine opportunities, and d) incorporate best practices guidelines and standards for physicians [4, 15]. Many of these recommendations overlap with others’ [32]. In addition to these critical features, future research should be designed with a pragmatic eye, adhering as closely as possible to the real world setting in which SMBG would be carried out by patients and utilized by patients and health care providers collaboratively. To date, no large-scale, pragmatic RCT has evaluated the impact of SMBG testing in patients with non-insulin treated T2DM in which a multi-dimensional approach to SMBG value management has occurred. This is the gap in knowledge that the MONITOR Trial will fill.

While SMBG may or not be worthwhile, effective SMBG, if it exists for non-insulin treated T2DM, appears to require that it be embedded within the context of patient education around the use and interpretation of glucose readings, provider awareness of the results of repeated testing, and collaborative use of this information at medical visits [33]. We would also argue for providing treatment algorithms to providers that are based on standard and accepted guidelines (such as the ADA guidelines) linked to SMBG report results [34]. This step facilitates the physician’s use of glucometer result reports and can be used by health care providers during clinic visits to better illustrate their concerns when talking to patients. Finally, we feel it is important to evaluate objectively and in a real world setting the possible additional benefits of personalized feedback for patients in the form of messages delivered via the glucometer based on patients’ current and recent SMBG patterns. By pointing out troubling patterns and rewarding results that are at goal, this aspect of the approach we are calling ‘enhanced feedback’ is akin to ‘mini consultations’ with a provider between routine clinic visits, which are generally 3–6 months apart.

## Abbreviations

HPLC: Certified high performance liquid chromotography
ITT: Intention to treat
NGSP: National Glycohemoglobin Standardization Program
NIT DM: Non-insulin treated diabetes mellitus
SMBG: Self-monitoring of blood glucose
T2DM: Type 2 diabetes mellitus
